# Genetic Evidence for SecY Translocon-Mediated Import of Two Contact-Dependent Growth Inhibition (CDI) Toxins

**DOI:** 10.1128/mBio.03367-20

**Published:** 2021-02-02

**Authors:** Allison M. Jones, Petra Virtanen, Disa Hammarlöf, William J. Allen, Ian Collinson, Christopher S. Hayes, David A. Low, Sanna Koskiniemi

**Affiliations:** a Department of Molecular, Cellular and Developmental Biology, University of California, Santa Barbara, Santa Barbara, California, USA; b Biomolecular Science and Engineering Program, University of California, Santa Barbara, Santa Barbara, California, USA; c Department of Cell and Molecular Biology, Uppsala University, Uppsala, Sweden; d School of Biochemistry, University of Bristol, Bristol, United Kingdom; University of Utah

**Keywords:** bacterial competition, type V secretion system, membrane potential, type V secretion, bacterial competition

## Abstract

The C-terminal (CT) toxin domains of contact-dependent growth inhibition (CDI) CdiA proteins target Gram-negative bacteria and must breach both the outer and inner membranes of target cells to exert growth inhibitory activity. Here, we examine two CdiA-CT toxins that exploit the bacterial general protein secretion machinery after delivery into the periplasm. A Ser281Phe amino acid substitution in transmembrane segment 7 of SecY, the universally conserved channel-forming subunit of the Sec translocon, decreases the cytotoxicity of the membrane depolarizing orphan10 toxin from enterohemorrhagic Escherichia coli EC869. Target cells expressing *secY^S281F^* and lacking either PpiD or YfgM, two SecY auxiliary factors, are fully protected from CDI-mediated inhibition either by CdiA-CT_o10_^EC869^ or by CdiA-CT^GN05224^, the latter being an EndoU RNase CdiA toxin from Klebsiella aerogenes GN05224 that has a related cytoplasm entry domain. RNase activity of CdiA-CT^GN05224^ was reduced in *secY^S281F^* target cells and absent in *secY^S281F^* Δ*ppiD* or *secY^S281F^* Δ*yfgM* target cells during competition co-cultures. Importantly, an allele-specific mutation in *secY* (*secY^G313W^*) renders Δ*ppiD* or Δ*yfgM* target cells specifically resistant to CdiA-CT^GN05224^ but not to CdiA-CT_o10_^EC869^, further suggesting a direct interaction between SecY and the CDI toxins. Our results provide genetic evidence of a unique confluence between the primary cellular export route for unfolded polypeptides and the import pathways of two CDI toxins.

## Abstract (precis)

Many bacterial species interact via direct cell-to-cell contact using CDI systems, which provide a mechanism to inject toxins that inhibit bacterial growth into one another. Here, we find that two CDI toxins, one that depolarizes membranes and another that degrades RNA, exploit the universally conserved SecY translocon machinery used to export proteins for target cell entry.

## INTRODUCTION

The Gram-negative bacterial cell envelope imposes a multitiered barrier to the movement of polypeptides, both into and out of the cell. Newly synthesized proteins destined for export travel via dedicated protein secretory pathways, passing through multicomponent assemblies that span part, or all, of the cell envelope. The majority of unfolded polypeptides transit through the Sec translocon, an inner membrane (IM)-embedded heterotrimeric complex formed by SecY, SecE, and SecG. Secreted proteins are generally translocated posttranslationally by the SecA motor protein, which uses ATP energy to deliver preprotein to SecYEG. This interaction alters the conformation of the lateral gate of SecY, unlocking and partially opening a central channel through SecY to facilitate secretion (1). In contrast, proteins destined for insertion in the IM contain transmembrane α-helices (TMH) that are transferred through the SecY lateral gate cotranslationally (2). The SecYEG core associates with additional accessory proteins, including YidC, which aids lateral insertion into the IM, and the YfgM/PpiD heterodimer, which binds to SecY at sites overlapping YidC and facilitates protein secretion under some conditions (3). Fully folded protein substrates cross the IM through the twin-arginine translocation translocase (4). Other proteins, including a number of bacterial toxins, exit the cell through highly specialized single- or double-membrane-spanning secretion systems (type 1 secretion system [T1SS] to T6SS) (5–9) or, in the case of colicins, via cell lysis (10, 11). Once exported, antibacterial toxins must traverse the envelope of target cells in the reverse direction to gain access to their molecular targets. Some toxins, such as those delivered by the T6SS, breach the outer membrane (OM) of recipient cells via the same macromolecular machinery through which they exited (12, 13). Others, such as the diffusible antibacterial colicin toxins from Escherichia coli, co-opt OM porins and transporters as well as transperiplasmic complexes that couple protein entry through the OM with energy from the proton motive force (PMF) (14, 15). The colicin nuclease ColE9 may utilize an inter-IM/OM membrane complex for transport of its nuclease domain into the cytosol (16) involving the IM protease FtsH and charge-dependent interactions with the IM (17).

Bacterial CDI toxins, in contrast to colicins, utilize a number of IM transport proteins to promote their translocation through the inner lipid bilayer (18). CDI toxins are encoded within the polymorphic C-terminal domains of filamentous CdiA proteins (ca. 40 to 100 nm), which are tethered to the OM and extend out from the cell surface (see Fig. S1 at https://datadryad.org/stash/share/9S_XL1c_3LqEvKDWKAAY8jnB44PNTbcLmUsf1tJdK0I) (19). CdiA is exported via the Sec-dependent secretion pathway and assembled on the surface of CDI^+^ cells by its T5SS partner and OM transporter, CdiB. CDI systems endow Gram-negative bacterial cells with growth inhibitory activity against closely related, nonimmune bacterial cells through the touch-dependent, receptor-mediated delivery of the CdiA toxin domain (19–21). Self-intoxication is prevented by expression of CdiI, which binds specifically to its cognate toxin and neutralizes it (22–25). Among E. coli CdiA proteins, multiple OM receptors have been identified: the BamA subunit of the OM β-barrel assembly machine complex, heterotrimers of OmpF/C osmoporins, and the nucleoside transporter Tsx (26–28). The receptor binding domains (RBDs) of these CdiAs are located centrally, but recent work indicates that CdiA folds such that the RBD is located at the tip of a thin hairpin fiber extending out from the cell surface, with the toxin-containing C-terminal half of CdiA localized within the periplasm (19). Receptor binding induces a series of conformational changes in CdiA that ultimately lead to transfer of the C-terminal toxin domain (CdiA-CT) into the periplasmic space of targets, where it is likely cleaved at or near the common (VE)NN sequence (19, 29). Genetic selections have identified specific target cell IM proteins (IMPs) that play critical roles at this step (18, 26, 30). For example, the glucose-specific transporter PtsG is required for CDI mediated by the C-terminal effector domain of CdiA from *E. coli* isolate EC3006 (CdiA^EC3006^), a tRNase (18). Target cells lacking PtsG are resistant to delivered CdiA-CT^EC3006^ but not to internally expressed toxin, suggesting that PtsG facilitates translocation of the toxin into the cytoplasm for access to the substrate. The ABC transporters MetI, RbsC, and YciB are required for import of the nuclease effector domains of CdiA^MHI813^, CdiA^Dd3937^, and CdiA_o11_^EC869^, respectively. Non-nuclease CDI toxins also require the presence of specific IMPs for activity (26). CdiA^EC93^ delivers a C-terminal effector domain that dissipates the PMF and decreases the ATP level within target cells, suggesting that it may form pores in the membrane (31). However, in contrast to pore-forming colicins that depolarize target cell membranes by spontaneously inserting into the lipid bilayer to form ion channels (32), CdiA-CT^EC93^ is not active against target cells that lack AcrB, the IM drug/proton antiporter component of the AcrAB-TolC multidrug efflux pump (26). The efflux pump activity is not required nor are its partners, since Δ*acrA* and Δ*tolC* target cells remain sensitive to CdiA-CT^EC93^. The mechanism(s) by which IMPs promote membrane insertion or translocation of CDI toxins is unknown. Here, we identify a single amino acid substitution (Ser281Phe), located deep within the channel of the universally conserved and essential protein export conduit, SecY, which confers resistance to the E. coli PMF-dissipating CdiA-CT_o10_^EC869^ toxin (one of 11 “orphan” CdiA-CT toxins encoded in the E. coli EC869 genome, downstream of the main CdiA-CT^EC869^). This is the first essential IMP identified with a role in CDI. Target cells carrying the *secY^S281F^* allele are protected from membrane depolarization and growth inhibition during competition co-culture with inhibitor cells despite receiving delivered CdiA-CT_o10_^EC869^. Notably, SecY^S281F^ does not appear to significantly affect protein translocation *in vitro*. The *secY^S281F^* mutation also provides partial protection against the tRNase activity of CdiA-CT^GN05224^, an EndoU RNase CDI toxin from Klebsiella aerogenes GN05224. Genetic selection for full toxin resistance identified two additional members of the SecY translocon, periplasmic chaperones YfgM and PpiD, that play roles in CDI toxin import. Taken together, our data indicate that CdiA-CT_o10_^EC869^ and CdiA-CT^GN05224^ toxins interact with the Sec translocon, effecting their insertion into and translocation across the IM, respectively.

## RESULTS

### A single amino acid substitution in transmembrane helix 7 of the protein translocase subunit SecY confers resistance to CdiA-CT_o10_^EC869^.

**FIG 1:**
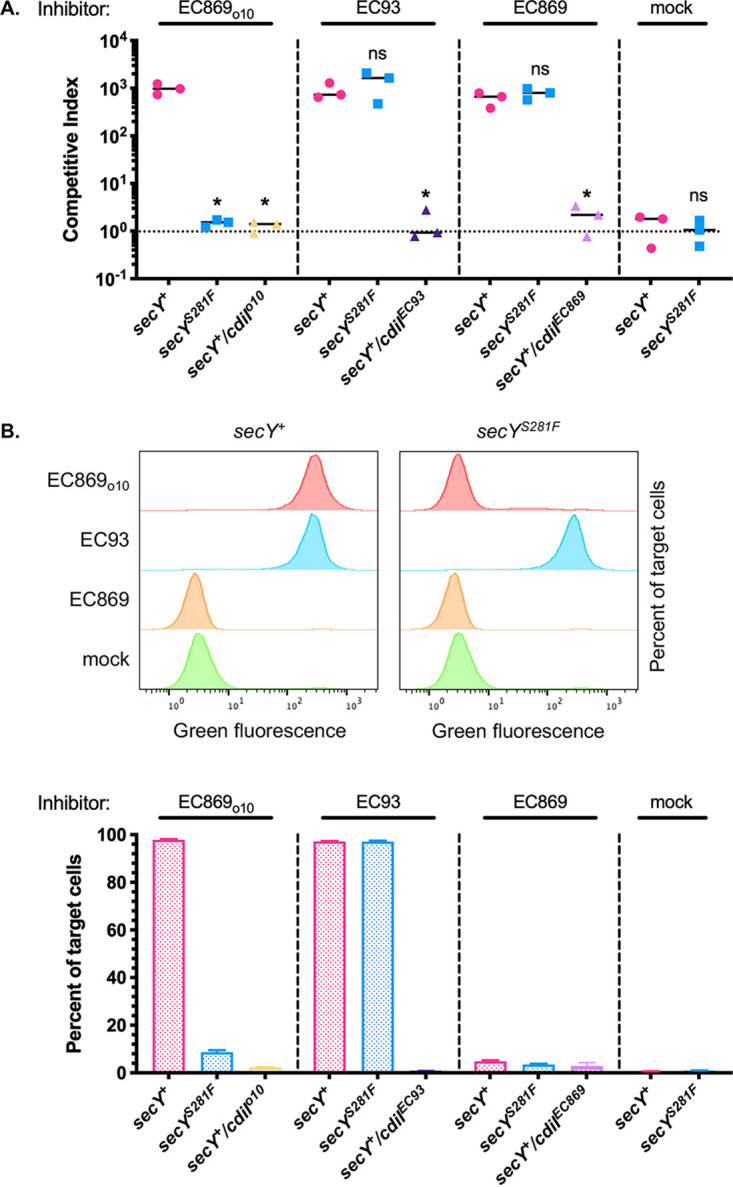
Evidence that SecY is a target of the PMF-dissipating CdiA-CT_o10_^EC869^. (A) CDI^+^ inhibitor cells delivering CdiA-CT_o10_^EC869^, CdiA-CT^EC93^, CdiA-CT^EC869^, and no toxin (mock) were co-cultured for 1 h with the indicated CDI^−^ target cells expressing dTomato from the chromosome at a 1:1 inhibitor-to-target cell ratio. Competitive indexes are shown on the *y* axis, calculated as the ratio of inhibitor cells to target cells at the end of the competition divided by the initial ratio. Results here, and for all competition growth experiments, are from at least three independent experiments, shown with the mean depicted by a solid line. Statistical significance was determined by Student’s *t* test (*, *P* < 0.05). (B) Upper panel, representative flow cytometry histograms showing incorporation of the membrane potential-sensitive dye DiBAC_4_(3) from the competition co-cultures in panel A. Lower panel, quantitation of membrane depolarization in target cells from competition co-cultures in panel A. Data are presented as the mean ± standard error of the mean (SEM) for three independent experiments. Colors in panels A and B are matched, showing the toxins delivered by inhibitor cells.

Previously, we showed that CdiA-CT_o10_^EC869^ inhibits the growth of E. coli target cells (29). However, the mechanism by which the toxin enters the cell and inhibits growth was not explored. CDI toxins require specific target cell factors for translocation and activity (18, 26, 33, 34). To identify such factors for CdiA-CT_o10_^EC869^, we used a genetic approach that we developed previously (34). Eleven independent pools of CDI-sensitive target cells were mutagenized by exposure to UV light and then subjected to three rounds of selection by co-culture with CDI^+^ inhibitor cells expressing a CdiA^EC93^-CT_o10_^EC869^ chimera to enrich for CDI-resistant (CDI^R^) mutants (29). Complementation analysis of one of the CDI^R^ mutants (M-Ec1) (see Materials and Methods; see also Table S1 at https://datadryad.org/stash/share/9S_XL1c_3LqEvKDWKAAY8jnB44PNTbcLmUsf1tJdK0I), identified a UCC (Ser)-to-UUC (Phe) missense mutation in *secY* that alters residue 281 in transmembrane helix 7 (TM7) of the protein (see Fig. S2 and Table S1 at the URL mentioned above). Target cells containing the *secY^S281F^* allele were significantly protected from growth inhibition during co-culture with inhibitor cells delivering CdiA-CT_o10_^EC869^ (compare *secY^S281F^* and *secY*^+^ target cells) (Fig. 1A). The CDI resistance observed is specific because *secY^S281F^* cells were sensitive to growth inhibition mediated by CdiA-CT^EC93^ and CdiA-CT^EC869^ (tRNase activity) (Fig. 1A). Subsequent sequence analysis of *secY* from four other independently isolated CDI^R^ mutants revealed a UCC (Ser)-to-UUU or UUC (Phe) mutation in all five lineages, supporting the importance of Phe281 in CDI resistance. Mutant isolates M-Ec2, M-Ec3, and M-Ec4 each contain additional missense mutations in *secY* (L423P, S68F, and V230L, respectively) (see Table S1 at the URL mentioned above). These three CDI^R^ mutants exhibited similar levels of resistance to CdiA-CT_o10_^EC869^ (see Fig. S3 at the URL mentioned above), indicating that the Ser281Phe substitution is primarily responsible for the CDI resistance phenotype. Notably, the *secY^S281F^* substitution mutation was detected in five independent UV-treated cell pools, and no other mutations were identified in the 11 pools analyzed. The frequency of a loss-of-function mutation, as well as the probability of having a mutation in any other gene, is far greater than that of having a single amino acid substitution five times in the essential *secY* gene product, encoded by two different base pair changes. Thus, our mutagenesis appears to be near saturating, suggesting that only mutations in the SecY^S281^ residue protect target cells from CdiA-CT_o10_^EC869^ intoxication.

The CdiI_o10_^EC869^ immunity protein that protects cells against CdiA-CT_o10_^EC869^ contains two predicted transmembrane helices. Colicins that inhibit bacterial growth by forming pores in the IM are neutralized by cognate immunity proteins that are integral IMPs (10), suggesting the possibility that CdiA-CT_o10_^EC869^ might disrupt the PMF. To test this hypothesis, we determined if the EC869_o10_ toxin dissipates the membrane potential, ΔΨ, via collapse of the proton gradient of target cells using the membrane potential-sensitive dye DiBAC_4_(3) [bis-(1,3-dibutylbarbituric acid)trimethine oxonol], which enters only depolarized cells. Target cells expressing dTomato from the chromosome were mixed with inhibitor cells expressing CdiA-CT_o10_^EC869^, and the percentage of dTomato fluorescent cells that incorporated the stain was investigated (see Fig. S4 at https://datadryad.org/stash/share/9S_XL1c_3LqEvKDWKAAY8jnB44PNTbcLmUsf1tJdK0I). Flow cytometric analysis indicated that CdiA-CT_o10_^EC869^ collapses the membrane potential, since almost all target cells (∼97%) incorporated the dye after co-culture with CDI^+^ inhibitor cells. Membrane depolarization was directly caused by the toxin, since cells expressing the CdiI_o10_^EC869^ immunity protein were protected (Fig. 1B). Target cells exposed to CdiA-CT^EC869^, which inhibits target cell growth by cleaving tRNA (29), did not incorporate dye (Fig. 1B), demonstrating that the DiBAC_4_ assay specifically detects depolarization of the membrane. Target cells containing the *secY^S281F^* mutation were fully protected from membrane depolarization by CdiA-CT_o10_^EC869^ but were not protected from the unrelated PMF-disrupting CdiA-CT^EC93^ toxin (Fig. 1B) (26), showing that *secY^S281F^*-mediated protection is specific. Taken together, these results indicate that CdiA-CT_o10_^EC869^ dissipates ΔΨ, which is necessary for ATP production and cell growth, and that target cells with the *secY^S281F^* allele are protected from this toxicity.

### Mutation in *secY* also protects against RNase activity of the CdiA-CT^GN05224^ nuclease toxin.

**FIG 2:**
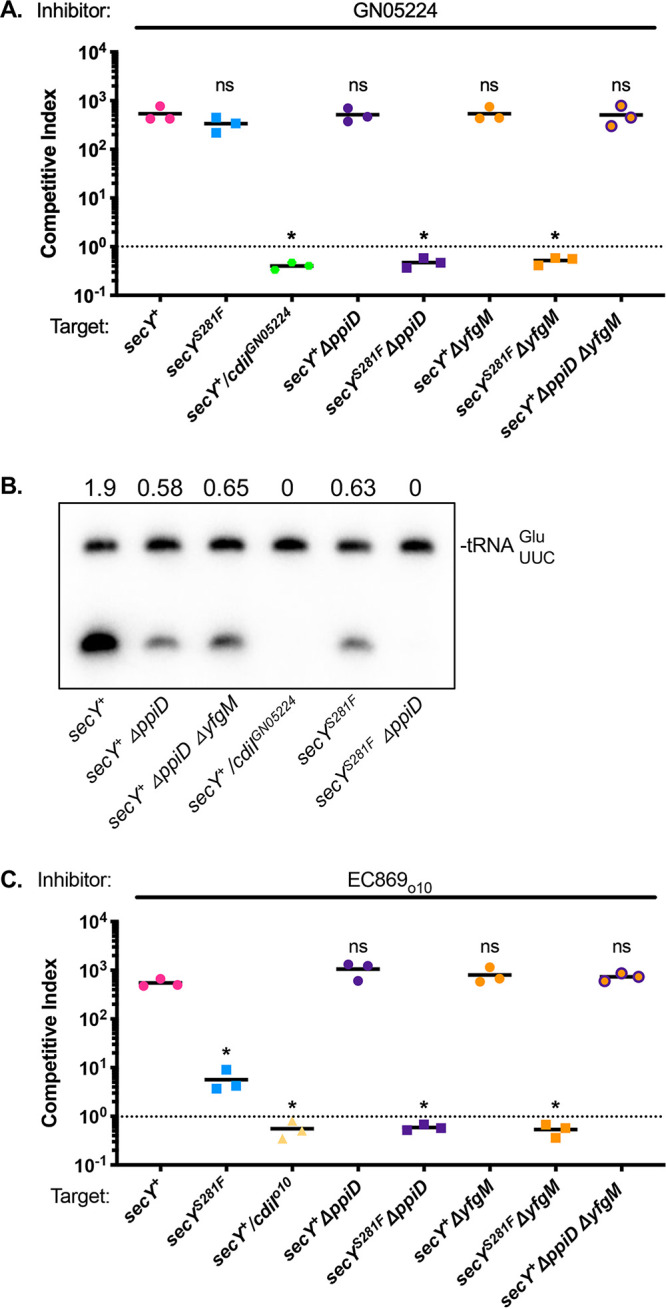
The SecY translocon accessory proteins PpiD and YfgM play roles in EC869_o10_ and GN05224 CDI toxin import. CDI^+^ inhibitor cells delivering CdiA-CT^GN05224^ RNase were co-cultured with the indicated CDI^−^ target cells at a 1:1 ratio. (A and B) Competitive indexes were calculated after 3 h of co-culture (A), or total RNA was extracted after 30 min of co-culture and used for Northern blot analysis of tRNA^Glu^ (B) The ratio of cleaved to uncleaved tRNA is indicated above each lane. (C) CDI^+^ inhibitor cells delivering the CdiA-CT_o10_^EC869^ were co-cultured with the indicated CDI^−^ target cells at a 1:1 ratio for 3 h. Statistical significance was determined by Student's *t* test (*, *P* < 0.05).

PMF-dissipating toxins such as CdiA-CT_o10_^EC869^ likely insert into the target cell IM, whereas nuclease toxins must cross the IM to access and cleave target nucleic acids in the cytosol. Previous work showed that specific target cell IMPs play critical roles in this process (18, 26, 30). For nuclease toxins, the interaction with specific IMPs is hypothesized to facilitate toxin translocation into the cytoplasm, and the genetic information that determines the specificity for a particular IMP is encoded within the cytoplasm entry domain (CED), located proximal to the C-terminal toxin domain (see Fig. S1 at https://datadryad.org/stash/share/9S_XL1c_3LqEvKDWKAAY8jnB44PNTbcLmUsf1tJdK0I) (18, 19). To determine if any CDI nuclease toxins exploit SecY to enter into the cytosol, we performed a blast search for CdiA-CT_o10_^EC869^ orthologs to identify predicted nuclease CdiA-CTs that contain entry domains similar to CdiA-CT_o10_^EC869^. Klebsiella aerogenes strain GN05224 contains a region (NCBI accession no. NZ_LDBZ01000036) encoding a CdiA-CT with an entry domain that shares 36% identity with CdiA-CT_o10_^EC869^ (Pfam database ID PF04829) (see Fig. S5A at the URL mentioned above) and a C-terminal toxin domain previously shown to cleave tRNA during competition co-cultures (35). A phylogenic analysis generated from the alignment of the CEDs (here chosen as the first 150 amino acids following the pretoxin VENN domain) of 20 CdiA-CTs indicates that CdiA-CT_o10_^EC869^ and CdiA-CT^GN05224^ cluster into two closely related subclades (see Fig. S5B, rectangular boxes, at the URL mentioned above). To examine toxin import through the IM specifically, we used CDI^+^ inhibitor cells expressing a CdiA^EC93^-CT^GN05224^ chimera (CDI^GN05224^) (35), leaving other known aspects of CdiA-mediated effector translocation such as receptor binding and processing unchanged. CDI^GN05224^ inhibitor cells outcompeted wild-type target cells as well as *secY^S281F^* target cells (Fig. 2A). We next examined RNase activity of the CdiA-CT^GN05224^ at an early time point during competition co-culture. Cleaved tRNA was detected in *secY^+^* target cells after 30 min of incubation with CDI^GN05224^ inhibitor cells (Fig. 2B, lane 1). Significantly less activity was observed in the *secY^S281F^* mutant than with *secY^+^* cells during that same time period (Fig. 2B, lane 5). Taken together, these results indicate that the *secY^S281F^* allele reduces entry of a CdiA-CT^GN05224^, although not enough to protect target cells from growth inhibition.

### SecY translocon accessory subunits PpiD and YfgM play roles in CDI toxin import.

Target cells expressing the *secY^S281F^* allele were not significantly resistant to CDI^+^ inhibitors delivering CdiA-CT^GN05224^, which could occur if CdiA-CT^GN05224^ interacts with different regions of SecY, or with another protein affected by the SecY mutation, for import. To further explore the CdiA-CT^GN05224^ import pathway, we sought to identify mutations that confer full resistance to CDI^GN05224^. Initially, we used a strategy similar to that which yielded CDI_o10_^EC869^-resistant mutants, but enrichment of CDI^GN05224^-resistant mutants was not observed in 16 independent mutant pools, suggesting that there are no nonessential proteins required for CdiA-CT^GN05224^ activity and no single amino acid substitutions that provide full resistance. Therefore, we instead used target cells carrying the *secY^S281F^* allele, which provides partial protection against tRNA cleavage mediated by CdiA-CT^GN05224^, for enrichment. After three cycles of enrichment, eight independent pools contained target cells fully resistant to CDI^GN05224^. To identify the mutations conferring CdiA-CT^GN05224^ resistance, the genomes of four independent mutant isolates were sequenced and compared to the parental strain. Three of the mutants contained null mutations in *yfgM*, and one mutant contained a mutation disrupting *ppiD* (see Table S1 at https://datadryad.org/stash/share/9S_XL1c_3LqEvKDWKAAY8jnB44PNTbcLmUsf1tJdK0I). Sequence analysis of the *yfgM* and *ppiD* alleles in the remaining four independent mutant isolates showed that all had frameshift or nonsense mutations in *yfgM* or *ppiD* (see Table S1 at the URL mentioned above). To directly assess the roles of the *yfgM* and *ppiD* genes in CDI resistance, we introduced in-frame deletions of *yfgM* and/or *ppiD* into *secY^+^* and *secY^S281F^* parental strains and performed competition co-cultures with inhibitor cells that deliver CdiA-CT^GN05224^ or CdiA-CT_o10_^EC869^. Target cells carrying the *secY^S281F^* allele with an in-frame deletion in *yfgM* (Δ*yfgM*) or *ppiD* (Δ*ppiD*) were fully resistant to inhibitor cells delivering both CdiA-CT toxins (Fig. 2A and C). In contrast, Δ*yfgM* and Δ*ppiD* mutants, both singly and in combination, were as sensitive to CDI as wild-type target cells. The CDI resistance observed was toxin specific, as none of the mutant target cells exhibited resistance to the CdiA-CT^EC869^ tRNase toxin (see Fig. S6 at the URL mentioned above). Target cells carrying both the *secY^S281F^* and Δ*ppiD* alleles are fully protected from CdiA-CT^GN05224^-mediated RNase activity (Fig. 2B), consistent with results showing full protection from growth inhibition (Fig. 2A). PpiD and YfgM are single-pass integral membrane proteins with large periplasmic domains and members of the periplasmic chaperone network (36–39). PpiD and YfgM interact with one another, forming a complex that associates with the Sec translocon (36, 40, 41). PpiD and YgfM are not required to activate CdiA-CT^GN05224^, which exhibits robust nuclease activity when purified and incubated with total E. coli RNA *in vitro* (35). Thus, these factors appear to play roles in facilitating CdiA-CT^GN05224^ translocation into the cytosol, where it has access to the RNA substrate. Because either of the genes coding for these proteins can be deleted in a *secY^S281F^* target cell to produce CDI resistance, our results indicate that the PpiD-YfgM complex plays a role in CDI toxin import.

### The *secY^S281F^* mutation blocks CDI at a step following toxin transfer to target cells.

**FIG 3:**
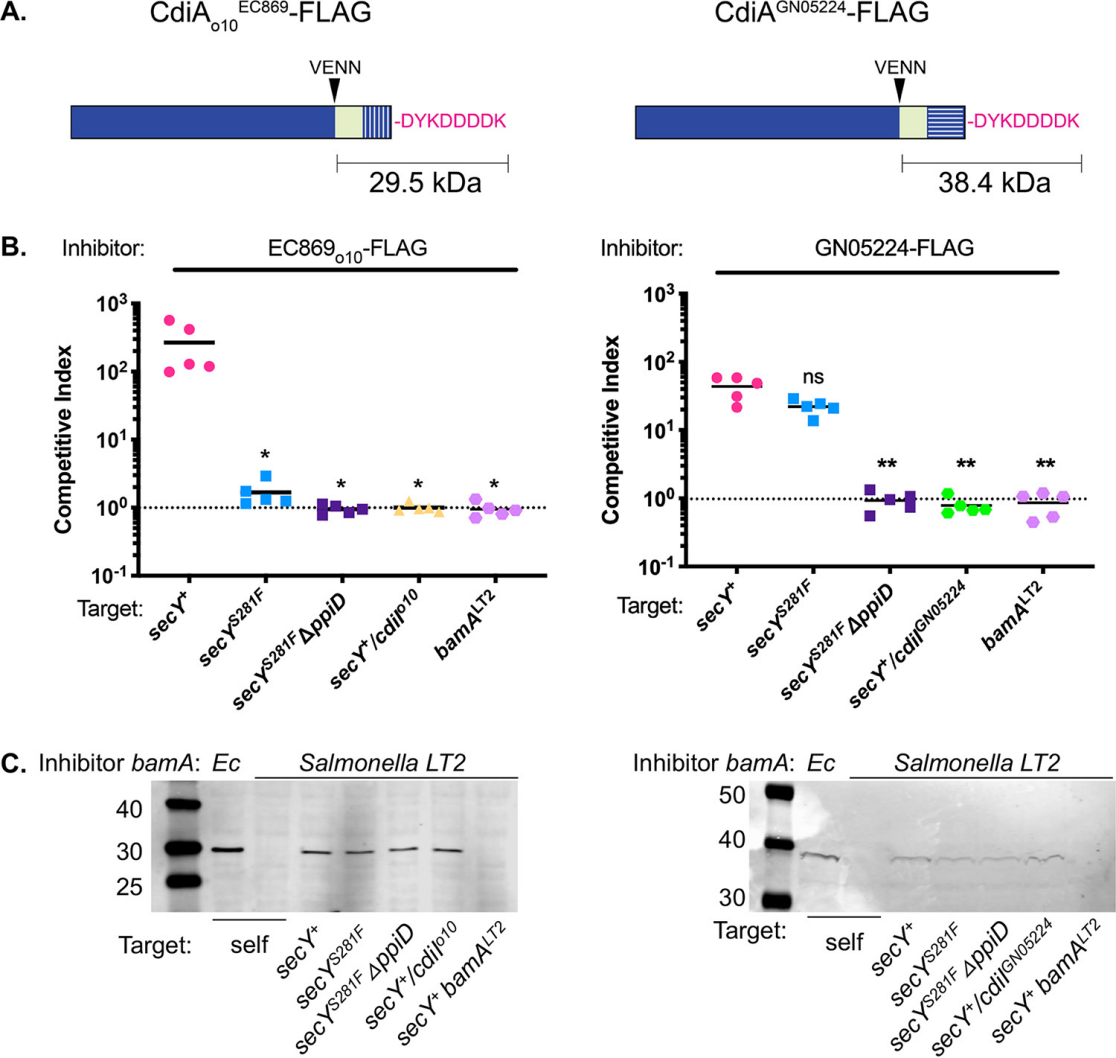
The *secY^S281F^* mutation blocks CDI toxin activity at a step following toxin delivery. Cellular delivery of FLAG epitope-tagged toxin domains in panels B and C were monitored by immunoblotting as described in Materials and Methods. (A) Diagrams of the constructs used in panels B and C. (B) CDI^+^ inhibitor cells delivering FLAG-tagged CdiA-CT_o10_^EC869-FLAG^ (left panel) or CdiA-CT^GN05224-FLAG^ (right panel) were co-cultured with the indicated CDI^−^ target cells at a 1:1 ratio for 1 h. Statistical significance was determined by Student's *t* test (*, *P* < 0.05; **, *P* < 0.01). (C) Western blots of the samples in panel B, using an anti-FLAG antibody. For these experiments, cells were mixed at high initial densities (OD_600_ = 2.8). The molecular weights of the full-length CdiA proteins are 324 kDa and 334 kDA for CdiA^EC93^-CT_o10_^EC869-FLAG^ and CdiA^EC93^-CT^GN05224-FLAG^, respectively.

PpiD and YfgM are periplasmic chaperones and may possibly affect CdiA-CT stability in the periplasm after delivery through the outer membrane but before entry into the inner membrane. Therefore, to monitor the fates of the EC869_o10_ and GN05224 CdiA-CT toxins post-delivery, we appended FLAG epitopes to their C-termini (Fig. 3A). FLAG-tagged toxins retained growth-inhibitory activity (Fig. 3B). For these experiments, CDI^+^ inhibitor cells and targets were mixed at a high initial cell density (optical density at 600 nm [OD_600_], 2.8) and co-cultured for 1 h. Toxin delivery to siblings was avoided by using CDI^+^ inhibitors expressing the heterologous OM receptor derived from Salmonella enterica serovar Typhimurium (BamA^LT2^), which blocks delivery of toxin (42). Immunoblotting with anti-FLAG antibody on the same samples as those shown in Fig. 3B revealed a band with the expected molecular weight of CdiA-CT_o10_^EC869^ (29.5 kDa) and CdiA-CT^GN05224^ (38.4 kDa) cleaved near the VENN sequence, only in cells capable of self-delivery in the absence of added targets (Fig. 3C, compare the 2nd and 3rd lanes from the left). Bands corresponding to the CdiA-CT toxins were also present in cell lysates from competition co-cultures in which susceptible (*secY^+^*), resistant (*secY^S281F^* and *secY^S281F^ΔppiD*), and immune (*secY^+^ cdiI_o10_^EC869^*) target cells were added, but not in cell lysates from competition co-cultures with *bamA^LT2^* target cells (Fig. 3C). These results indicate that the *secY^S281F^* mutation does not prevent CdiA-CT_o10_^EC869^ transfer through the OM into cells or cleavage near the VENN sequence (19). In addition, both CdiA-CTs were detected in target cells lacking PpiD (Fig. 3C), suggesting that the role of PpiD/YfgM is not to protect the toxins from proteases but to facilitate transport into or through the inner membrane. Together, these results indicate that the CEDs of CdiA-CT_o10_^EC869^ and CdiA-CT^GN05224^ likely interact directly with a cytoplasmic membrane component(s) of the toxin import pathway involving SecY, YfgM, and PpiD. Because CdiA toxin processing and C-terminal cleavage occur in *secY*, *yfgM*, and *ppiD* mutant backgrounds (Fig. 3C), it seems likely that processing of CdiA toxins occurs during transport through the OM or immediately after, although it is possible that cleavage could occur at the IM.

### SecY^S281F^ does not significantly alter general protein translocation.

The *secY^S281F^* mutation was isolated from multiple independent rounds of resistance selection, and two different synonymous single nucleotide polymorphisms (SNPs) were identified, strongly suggesting that the S281 residue is crucial for the CDI resistance. However, it is still possible that the mutation alters the abundance of another essential protein in the inner membrane. To determine if the *secY^S281F^* mutant indirectly affected protein secretion, we tested the *in vitro* protein secretion activity of the translocons encoded by the wild-type and mutant isolates. We found that the SecY^S281F^EG variant was fully capable of OmpA secretion and only marginally slower than the native version (see Fig. S7A at https://datadryad.org/stash/share/9S_XL1c_3LqEvKDWKAAY8jnB44PNTbcLmUsf1tJdK0I). Immunoblot analyses indicated that no significant differences in protein levels could be detected in the abundance of SecE or SecG between the wild type and the *secY^S281F^* mutants (see Fig. S7B at the URL mentioned above). As a control, BamA receptor levels were compared between the wild type and the *secY^S281F^* mutants. No significant differences in the abundance of BamA could be observed between the strains, indicating that the SecY-dependent export of BamA to the OM is unaffected (see Fig. S7B at the URL mentioned above). Lastly, we performed mass spectrometry on envelope-enriched fractions of the wild type and the *secY^S281F^* mutant to investigate if the mutation resulted in global changes of the protein composition of the envelope. We could detect only 30% of the essential proteins secreted by SecY (as the majority of proteins detected were cytosolic [791 out of 972]) (see Table S2 at the URL mentioned above), but none of these proteins changed significantly between the wild type and the *secY^S281F^* mutant (see Fig. S8 at the URL mentioned above), suggesting that the SecY^S281S^ mutant does not have a general deficiency in protein secretion. Taken together, these results support the hypothesis that the *secY^S281F^* mutation affects CDI toxin import directly.

### Further genetic analysis addressing the role of SecY in CDI toxin import.

**FIG 4:**
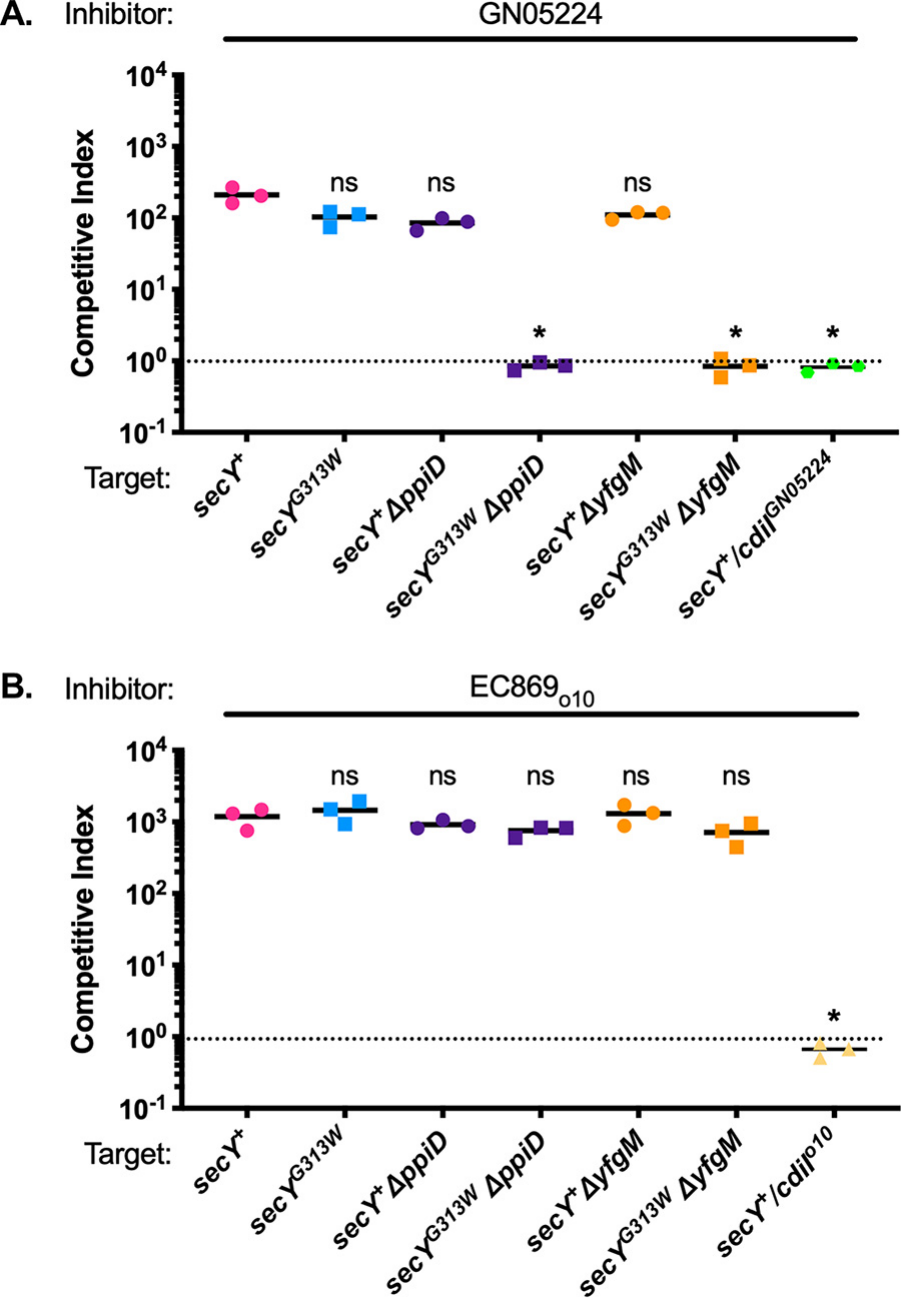
Identification of additional residues in SecY that function in import of the GN05224 toxin in the absence of PpiD and YfgM accessory factors. CDI^+^ inhibitor cells expressing the CDI_o10_^EC869^ (A) or CDI^GN05224^ (B) systems were mixed at a 1:1 ratio and co-cultured for 3 h with the indicated CDI^−^ target cells with the indicated mutations. Statistical significance was determined by Student's *t* test (*, *P* < 0.05).

Attempts to detect a direct interaction between CDI toxins and SecY with cross-linking experiments utilizing the FLAG epitope-tagged toxins followed by immunoblot analysis were unsuccessful, potentially due to the transient nature of the CDI toxin-IMP interaction. Therefore, we took a genetic approach to isolate mutations that provide specific resistance to CdiA-CT^GN05224^. These mutations could potentially identify another protein required for CdiA-CT^GN05224^ transport through the inner membrane, or mutations in SecY that are allele specific for the CdiA-CT^GN05224^ CED domain, which would indicate a direct interaction. We performed mutagenesis and enrichment in competition co-cultures using CDI^GN05224^ inhibitor cells and either *ΔppiD* or *ΔyfgM* target cells. All resulting CDI-resistant mutants contained single missense mutations in *secY* (see Table S1 at https://datadryad.org/stash/share/9S_XL1c_3LqEvKDWKAAY8jnB44PNTbcLmUsf1tJdK0I). Three of the mutants were resistant to CdiA-CT^GN05224^ but not to CdiA-CT_o10_^EC869^, thus showing allele specificity. Whole-genome sequencing showed that none of these mutants carried additional mutations in essential genes (see Table S3 at the URL mentioned above), suggesting that the G313W, L386P, and G403R amino acid substitutions in SecY provided resistance specifically to CdiA-CT^GN05224^. To verify this, we replaced *secY^+^* with the secY^G313W^ allele in wild-type and *ΔppiD* or *ΔyfgM* MG1655 E. coli cells and confirmed that a glycine-to-tryptophan substitution at residue 313 (G313W) in either *ΔppiD* or *ΔyfgM* backgrounds (but not in the wild type) was sufficient to provide resistance to CdiA-CT^GN05224^ (Fig. 4A) but not to CdiA-CT_o10_^EC869^ (Fig. 4B). Viewed from the periplasm, these residues are in a ring in the resting state (see Fig. S9 at the URL mentioned above). Gly313 appears to be especially dynamic during protein translocation, undergoing a conformational change between the resting and the unlocked (43). Together, these results strongly support the hypothesis that the *secY^S281F^* and *secY^G313W^* mutations affect CDI toxin import directly. The alternative hypothesis, i.e., that each of the *secY* mutations affects the abundance of a different essential inner membrane protein and that these hypothetical proteins were not detected in our analysis of the SecY secreted envelope proteins (see Fig. S8 at the URL mentioned above), seems very unlikely.

## DISCUSSION

Following translocation through the OM, CDI toxins are cleaved at or near their conserved (VE)NN sites and must additionally cross or enter the IM barrier to elicit growth inhibitory activity (19). Our results here shed light on this latter step in the CDI pathway, indicating that the CdiA-CT^GN05224^ toxin which cleaves tRNAs in the cytosol and the CdiA-CT_o10_^EC869^ toxin that permeabilizes membranes, each utilize components of the SecY translocon for their import. Visualization of FLAG epitope-tagged CdiA-CT_o10_^EC869^ or CdiA-CT^GN05224^ indicated that toxin processing and transfer into the periplasm occurred normally in the *secY^S281F^* mutant background, but translocation into or through the IM was blocked. Genetic evidence indicates that SecY plays a direct role in CDI import since resistance of the *secY^G313W^* mutant to CdiA-CT^GN05224^ was allele specific (in *ΔppiD* or *ΔyfgM* backgrounds).

The *secY^S281F^* mutation was sufficient to provide full resistance against the CdiA-CT_o10_^EC869^ toxin but not the CdiA-CT^GN05224^ toxin. This could be explained if the S281 residue is not only important for recognizing the CED domain of the incoming toxin for transport but also facilitates entry of the peptide into the membrane. Thus, full resistance is achieved for the membrane ionophore toxin CdiA-CT_o10_^EC869^ but not the tRNase toxin CdiA-CT^GN05224^, which is still able to enter the cell, although less efficiently. Another possibility is that the number of toxins required for toxicity is different for the two toxins and that more toxin molecules are required to form a pore than to function as a tRNase in the cytosol. Thus, if the mutation simply lowers the rate at which toxins enter the membrane or cell, the steady-state level of toxins might become too low for toxicity.

During general protein secretion, the SecYEG translocon associates with distinct binding partners, forming multimeric allosteric ensembles whose member constituency depends on the translocating substrate (1, 44, 45). By engaging substrate and SecYEG, the essential and auxiliary SecY-associated factors, which include SecA, SecDF-YajC, YidC, and PpiD-YfgM, modulate the activity of the translocon to increase the efficiency of protein export and IMP biogenesis. Although YfgM and PpiD appear to play roles in CDI toxin import in the *secY^S281F^* background, deletion of the genes coding for these proteins had little to no effect on CDI toxin sensitivity of *secY*^+^ target cells. YfgM and PpiD thus appear to play accessory roles with regard to SecY-mediated translocation of these toxins, analogous to their accessory roles in the general secretory pathway. During protein translocation, the large periplasmic domain of PpiD interacts with translocating polypeptides as they exit SecY (46), facilitating the release of newly secreted peptides from the Sec translocon and interaction with SurA and SkpA as part of a chaperone relay network (47). YfgM/PpiD do not appear to function to protect CDI toxins from proteases, as the delivered CdiA-CTs were found to be equally stable in both the wild-type and mutant cells. Therefore, it is possible that YfgM and PpiD might help guide CDI toxins to SecY, maintaining the toxin in an unfolded, translocation-competent state during entry into the lipid bilayer. Another possibility is that the PpiD-YfgM complex alters the lipid environment at the SecY lateral gate, facilitating toxin entry or translocation. Recent evidence indicates that PpiD and YidC use overlapping binding sites on SecY and that the PpiD/YfgM chaperone complex is the most prominent interaction partner of SecYEG (3). These results might explain why multiple *ppiD* and *yfgM* mutants were identified in our screen whereas no mutations in *yidC* were found.

Transient interactions with accessory factors as well as specific mutations in *secY*, such as the *prlA* mutations (48), alter the conformational dynamics of the channel and hence the functioning of the Sec translocon. Protein localization (*prl*) mutations in *secY* are thought to promote an activated, translocation-competent conformational state in the absence of signal sequence binding, permitting the export of preproteins with defective or missing signal peptides (41–46, 48). For example, *prlA4* suppresses the growth defect of a *lamB14D* mutant lacking a functional maltodextrin transporter signal sequence in cells plated on dextrin minimal agar. Cells carrying the *secY^S281F^* allele that confer CDI resistance have no apparent defects in protein export. Unlike *prlA* mutant cells, *secY^S281F^* cells were unable to export signal sequence-defective LamB polypeptides (see Fig. S10A at https://datadryad.org/stash/share/9S_XL1c_3LqEvKDWKAAY8jnB44PNTbcLmUsf1tJdK0I). The S281F substitution does not appear to trigger the channel for protein export, and the amino acid substitutions encoded by the *prlA* alleles we tested that do induce a translocation-ready conformation of SecY did not provide protection against the EC869_o10_ and GN05224 CDI toxins (see Fig. S10B at the URL mentioned above). Together, these observations indicate that SecY functions in toxin import are different from its normal protein export functions.

We recently showed that CdiA toxin processing via C-terminal cleavage occurs only after binding to the cognate outer membrane receptor (19). Nuclease CdiA-CTs enter the periplasm upon receptor binding regardless of the metabolic state of the receiving cell but require the PMF for transport through the IM into the cell cytosol (29). Our data here show that C-terminal cleavage of CdiA occurs in the *secY^S281F^* mutant background in which toxin import is blocked and the released C-terminal toxin is stable enough to detect. Thus, toxin cleavage seems to occur independently from translocation across the IM, indicating that translocation across the inner and outer membranes can be disconnected. In contrast to CDI, colicins require the PMF for translocation across the OM, and processing of the nuclease domain of colicins ColE2-E3 and ColD occurs during transport through the inner membrane (49, 50). Our results indicate that CdiA-CT transport is distinct from these colicins, but it cannot be ruled out that the cleavage occurs during transport through the inner membrane but that the CdiA-CTs become stalled in the IM during the translocation event in mutant cells.

CdiA-CT_o10_^EC869^ and CdiA-CT^GN05224^ appear to utilize at least part of the SecY translocon for import through the IM of target cells. One possible mechanism is that toxins follow a retrograde pathway through the SecYEG channel, interacting with TM7 of SecY. If toxins are delivered into targets in the unfolded state, they may be able to thread into the export channel by using energy from the proton gradient, which we showed previously is required for toxin import (29). Recently, Corey et al. demonstrated that membrane-bound cardiolipin, an anionic phospholipid, is required for the PMF enhancement of protein translocation through the SecYEG translocon (51). If the toxins follow a retrograde transport pathway, however, the PMF component they harness must be distinct from that of protein export, because CdiA-CT_o10_^EC869^ and CdiA-CT^GNO5224^ are fully active against cells lacking cardiolipin (see Fig. S11 at https://datadryad.org/stash/share/9S_XL1c_3LqEvKDWKAAY8jnB44PNTbcLmUsf1tJdK0I). Alternatively, CDI toxins might travel through an encapsulated lipid microenvironment generated by the arrangement of individual subunits within Sec translocon subassemblies. Recent small-angle neutron scattering of the YidC-SecYEG-SecDF holotranslocon confirmed a central lipid-filled core located between SecY and YidC at the lateral gate of SecY (52). The PpiD-YfgM complex, which is the predominant interacting partner of the SecYEG translocon, uses overlapping SecY-binding sites to YidC and thus could affect the lipid environment at the lateral gate similarly (3). Previous work showed that cholera toxin binding to its receptor perturbs lipid packing, and these changes in lipid “texture” are transmitted to the inner leaflet of the membrane (53). This type of lipid alteration, possibly in combination with steric changes in the Sec translocon subassemblies during protein export, might aid peptide transit at the lipid-SecYEG protein interface. Further investigation is required to determine whether the substitutions in SecY that confer CDI resistance are in residues that directly contact the importing toxin polypeptide or indirectly affect the conformational plasticity of SecY, abrogating its function in toxin import.

Some bacterial pathogens express mammalian cell-targeting AB toxins that, like the GN05224 CDI toxin, must be imported into the cytosol for activity. These toxins are routed via retrograde transport to the endoplasmic reticulum (ER) before entering the cytosol (54). Once in the lumen of the ER, the catalytic A subunit of several AB toxins engage components of the ER-associated degradation (ERAD) pathway, which usually targets misfolded proteins in the ER to the cytosol for proteolysis. AB toxins of this subgroup appear to hijack membrane-embedded channels, possibly including the Sec61 (SecY homolog) translocon (55). Notably, these AB toxins interact with ERAD-associated chaperones, which disassemble the AB subunits, unfold the catalytic A domain, and maintain it in a translocation-competent state (56–59). Thus, the co-opting of cellular protein translocation-associated chaperones and channels by bacterial toxins appears to occur in both the prokaryotic and eukaryotic kingdoms.

## MATERIALS AND METHODS

### Bacterial and CDI competition co-cultures.

Bacterial strains are shown in Table S4 at https://datadryad.org/stash/share/9S_XL1c_3LqEvKDWKAAY8jnB44PNTbcLmUsf1tJdK0I. Bacteria were grown at 37°C in LB medium or on LB agar unless otherwise noted. Media were supplemented with antibiotics at the following concentrations: ampicillin (AMP), 150 μg ml^−1^; kanamycin (KAN), 40 μg ml^−1^; chloramphenicol (CAM), 12.5, 34, or 60 μg ml^−1^; and spectinomycin (SPEC), 50 μg ml^−1^.

For liquid competition co-cultures, inhibitor and target cells were grown to mid-logarithmic phase and then mixed at a 1:1 ratio in medium without antibiotics. Co-cultures were incubated for the times indicated (1 h or 3 h) at 37°C with shaking at 225 rpm in baffled flasks. Viable inhibitor and target cells were counted as CFU on selective LB agar. Spin down competition co-cultures were used when target cells had different growth rates (see Fig. S5 and S10 at the URL indicated above). For spin down competition co-cultures, inhibitor and target cells were grown to mid-log phase and then mixed at a 1:1 ratio in medium without antibiotics in a 1.5-ml Eppendorf tube. Cells were centrifuged at 300 × *g* for 2 min. Cell pellets were then resuspended. Viable inhibitor and target cells were enumerated as CFU on selective LB agar. The competitive index (CI) was calculated as the ratio of inhibitor to target cells after centrifugation divided by the initial inhibitor-to-target cell ratio.

### Isolation of resistant mutants.

To generate CDI-resistant mutants, target cells were UV mutagenized and selected for as previously described (34). The CdiA^EC93^-CT_o10_^EC869^ and CdiA^EC93^-CT^GN05224^ chimeras bind to the OM receptor BamA. To avoid isolating mutations in *bamA*, we introduced a *bamA^+^* multicopy plasmid (see Table S5 at the URL mentioned above) into target cells prior to mutagenesis. Each mutant pool was subjected to selection by co-culture with E. coli EPI100 inhibitor cells carrying pCH10166 or pDAL8914. The surviving colonies were collected and subjected to two more rounds of coculture selection. After three rounds of selection, individual colonies were picked and tested for resistance. Mutations in resistant colonies were determined through complementation with cosmid libraries or through whole-genome sequencing. For detailed analysis of these methods, see the appendix at https://datadryad.org/stash/share/9S_XL1c_3LqEvKDWKAAY8jnB44PNTbcLmUsf1tJdK0I.

### Membrane potential measurements.

Overnight cultures of target and inhibitor cells were diluted 1:100 into fresh LB broth and grown to an OD_600_ of 1.0 (or approximately 5 × 10^7^ CFU/ml) and mixed at a ratio of 1:1. After 1 h of co-culture, an aliquot of 150 ml was moved to a 1.5-ml Eppendorf tube, and 10 mg/ml of DiBAC_4_(3) was added. Cells were incubated at 37°C with shaking for another 30 min. Single-cell fluorescence was measured using a MACSQuant VYB (Miltenyi Biotec). DiBAC_4_(3) was excited at 488 nm, and its fluorescence was measured on the B1 channel (filter 525/50 nm). dTomato was excited at 561 nm, and its fluorescence was measured on the Y2 channel (filter 615/20 nm). A total of 100,000 events were collected. Raw flow cytometry data analysis was performed with the FlowJo data analysis software program (FLOWJO LLC, Ashland, OR).

Detailed descriptions of the construction of E. coli strains (Table S4), plasmids (Table S5), oligonucleotides (Table S6), maltodextrin selection, cosmid library construction, complementation, genome sequencing, immunoblotting, and RNA analysis are provided in the appendix at the URL mentioned above.

### Data availability.

All data and methods discussed in this paper are available in the main text and in the appendix at https://datadryad.org/stash/share/9S_XL1c_3LqEvKDWKAAY8jnB44PNTbcLmUsf1tJdK0I. Raw data for growth competition experiments are shown in Table S7, and all supplemental information referenced in this paper is available through Dryad at the URL mentioned above.

Detailed protocols are available from S.K. or D.A.L. upon request.
